# Analysis of maize PAL pan gene family and expression pattern under lepidopteran insect stress

**DOI:** 10.3389/fpls.2025.1651563

**Published:** 2025-09-01

**Authors:** Tonghan Wang, Yaohui Zheng, Lu Sun, Minghui Guan, Ying Hu, Haibing Yu, Degong Wu, Junli Du

**Affiliations:** ^1^ Engineering Technology Institute of Maize Breeding in Anhui Province, Chuzhou, China; ^2^ College of Agriculture, Anhui Science and Technology University, Chuzhou, China; ^3^ College of Resources and Environment, Anhui Science and Technology University, Chuzhou, China; ^4^ Anhui Province International Joint Research Center of Forage Bio-breeding, Chuzhou, China

**Keywords:** maize pan-genome, phenylalanine ammonia-lyase, structural variation, selection pressure, insect stress

## Abstract

**Introduction:**

Phenylalanine ammonia-lyase (PAL), as the rate-limiting enzyme in plant phenylpropanoid metabolism, catalyzes the conversion of L-phenylalanine to trans-cinnamic acid and plays a pivotal role in plant-insect resistance mechanisms.

**Methods:**

Utilizing a maize pangenome constructed from 26 high-quality genomes, we systematically identified the *ZmPAL* gene family members. Evolutionary pressure and structural variation (SV) analyses were conducted, alongside reanalysis of publicly available RNA-seq datasets under lepidopteran stress conditions. Temporal expression patterns were further validated via qRT-PCR.

**Results:**

This investigation identified 29 *ZmPAL* genes, comprising 7 core, 2 near-core, 12 dispensable, and 8 private genes, revealing substantial limitations of single-reference genome-based studies. Evolutionary analysis indicated positive selection of *ZmPAL8* in specific germplasms, while SV-affected *ZmPAL5* exhibited significantly divergent expression patterns. Conserved expression profiles were observed among *ZmPAL* members under diverse lepidopteran stresses. Temporal-specific regulation was established: *ZmPAL7, ZmPAL10*, and *ZmPAL23* dominated early defense responses, whereas *ZmPAL10* and *ZmPAL23* maintained predominance during mid-late phases.

**Discussion:**

This pangenome-based study provides novel insights into PAL-mediated phytoprotective mechanisms against lepidopteran pests and establishes a theoretical framework for understanding maize's molecular adaptation to biotic stressors.

## 1 Introduction

Plant metabolic systems are categorized into primary and secondary pathways, with the phenylpropanoid pathway constituting one of three principal secondary metabolic routes (Crozier et al., 2006; Kumar et al., 2023; Salam et al., 2023; Han et al., 2025). Initiated by PAL, this pathway catalyzes the first enzymatic conversion of phenylpropanoid compounds to generate intermediates including coumaric acid, ferulic acid, and sinapic acid (MacDonald and D’Cunha, 2007; Levy et al., 2018; Zheng et al., 2024). Through sequential enzymatic conversions, these precursors form coumarins, chlorogenic acids, and phenylpropanoyl-CoA esters, ultimately yielding diverse phenylpropanoids such as flavonoids, lignin, cinnamates, and alkaloids (Rhodes and Wooltorton, 1976; Lavhale et al., 2018; Zhou et al., 2025). The phenylpropanoid pathway produces abundant phenolic derivatives that serve as precursors for phytohormones, anthocyanins, phytoalexins, and structural polymers (Dong and Lin, 2021). Particularly, phenylpropanoid derivatives play critical functions in plant defense mechanisms against phytophagous insects and microbial pathogens through their roles in physical barrier formation and antimicrobial compound biosynthesis (Dixon et al., 2002; Dong and Lin, 2021).

Phenylalanine ammonia-lyase (PAL; EC 4.3.1.5), functioning as the pivotal rate-limiting enzyme in phenylpropanoid metabolism (Vogt, 2010), catalyzes the deamination of L-phenylalanine to trans-cinnamic acid, thereby initiating biosynthetic cascades that yield critical secondary metabolites including flavonoids, anthocyanins, and lignin (Liu et al., 2023b; Qiu et al., 2024). This enzyme exhibits ubiquitous presence across photosynthetic organisms, being phylogenetically conserved in plants, fungi, yeasts, and algae, though notably absent in metazoans (Emiliani et al., 2009; Kawatra et al., 2020). Molecular characterization of PAL isoforms has been documented in numerous taxonomically diverse species, including but not limited to *Arabidopsis thaliana* (Kawatra et al., 2020), *Brassica napus* (Zhang et al., 2023), *Populus tremula* (Subramaniam et al., 1993), *Sorghum bicolor* (Pant and Huang, 2022), *Musa acuminata* (Wuyts et al., 2006), *Solenostemon scutellarioides* (Zhu et al., 2015), *Oryza sativa* (Yu et al., 2018), *Triticum aestivum* (Feduraev et al., 2020), and *Juglans regia* (Xu et al., 2012; Yan et al., 2019), demonstrating its evolutionary significance in secondary metabolic processes.

The PAL gene family members function as key regulators in plant developmental processes (Elkind et al., 1990; Ramzan et al., 2023). In *Arabidopsis thaliana*, *AtPAL1*, *AtPAL2*, and *AtPAL4* demonstrate tissue-specific expression patterns, with preferential accumulation in stems and seeds (Cochrane et al., 2004). Similarly, 11 out of 12 identified *ClPAL* genes in watermelon (*Citrullus lanatus*) exhibit substantial transcriptional activity in stems and floral organs (Dong and Shang, 2013). Beyond developmental regulation, PAL isoforms mediate plant responses to diverse biotic and abiotic stresses (Wang et al., 2025). For instance, *AtPAL1* and *AtPAL2* upregulation under nitrogen deficiency and thermal fluctuations drives flavonoid accumulation in Arabidopsis (Ni et al., 2008c). Maize cultivars exposed to heat stress show elevated *ZmPAL2* and *ZmPAL11* expression, correlating with enhanced PAL enzymatic activity and concurrent increases in total phenolics/flavonoids (Wu et al., 2020). Conversely, AevPAL1 overexpression in bread wheat (*Aegilops variabilis*) confers resistance against cereal cyst nematode (*Heterodera avenae*) (Zhang et al., 2021), while Puccinia striiformis infection differentially modulates 25 wheat PAL homologs (11 upregulated, 14 downregulated) (Sørensen et al., 2016). Functional studies in rice reveal that *OsPAL6* and *OsPAL8* overexpression elevates lignin and salicylic acid biosynthesis, enhancing resistance to brown planthopper (*Nilaparvata lugens*) (He et al., 2019; Yang et al., 2024). Notably, PAL induction exhibits systemic signaling properties - both herbivore-damaged and adjacent undamaged cotton (*Gossypium hirsutum*) and maize seedlings display synchronized PAL activation, suggesting interspecies communication through phenylpropanoid-mediated defense priming (Lv et al., 2016; Hu et al., 2022).

Maize (*Zea mays* L.), a cornerstone crop in global food security, plays pivotal roles in human nutrition, livestock feed production, industrial applications, and bioenergy development (Lu et al., 2018; Yan and Tan, 2019; Yin et al., 2024). Escalating challenges from climate change and ecological shifts have intensified crop vulnerability to phytophagous pests, particularly lepidopteran species that severely compromise maize productivity (Zeng et al., 2020; Bebber, 2021). Key defoliators including fall armyworm (*Spodoptera frugiperda*, FAW), Asian corn borer (*Ostrinia furnacalis*, ACB), and beet armyworm (*Spodoptera exigua*, BAW) inflict systemic damage through folivory, stem boring, and ear feeding during critical growth stages (Tzin et al., 2017; Lu et al., 2024; Zhao et al., 2024). This biotic stress necessitates urgent identification of stress-resistance genes and development of resilient cultivars - strategies crucial for yield optimization, quality enhancement, and climate-smart agricultural adaptation.

Investigation of *ZmPAL* genes holds significant agricultural importance given phenylpropanoids’ critical roles in phytophagous insect resistance (Li et al., 2021; Ramaroson et al., 2022). While previous studies have characterized maize PAL family members employing conventional single-reference genome approaches, systematic analyses under lepidopteran herbivory remain unexplored (Wu et al., 2020). Traditional gene family identification methods, constrained by single-genome frameworks, inherently fail to detect non-reference members existing in other germplasm (Bi et al., 2025; Tong et al., 2025). The maize pangenome resource established by Hufford et al., comprising 26 high-quality genomes with extensive presence-absence variations (PAVs) and structural variations (SVs), provides unprecedented resolution for pan-genomic studies (Funk and Zahn, 2021; Hufford et al., 2021). Leveraging this pangenomic architecture, researchers have successfully delineated multiple pan-gene families including *TPS* (Sun et al., 2023), *ARF* (Man et al., 2025), and *Ann* (Liu et al., 2025), demonstrating the framework’s capacity to reveal previously undetected genetic diversity. This approach enables comprehensive investigation of gene family evolution and functional diversification across maize germplasm.

This study systematically characterized the *ZmPAL*s pangenome family across 26 maize genomes, profiling presence-absence variations, selection pressures, cis-regulatory elements, and structural motifs. By integrating publicly available RNA-seq datasets with qRT-PCR validation, the expression dynamics of *ZmPAL* members under lepidopteran infestation were comprehensively deciphered. The findings yield foundational insights into *ZmPAL*-mediated molecular responses to lepidopteran stressors while establishing a molecular framework for understanding regulatory mechanisms during herbivore challenges. These results provide actionable genetic targets for developing insect-resistant maize cultivars through molecular breeding strategies, addressing critical agricultural demands for sustainable pest management solutions.

## 2 Materials and methods

### 2.1 Plant material preparation and insect rearing

All experiments were conducted at the Plant Growth Facility of Anhui Science and Technology University (32°55’N, 117°23’E), located in Chuzhou, Anhui Province, China. The facility maintains controlled-environment chambers with standardized conditions for photoperiod, temperature, and humidity control. The maize inbred line B73 was cultivated in a controlled-environment growth chamber using a specific substrate composed of a sterilized potting mixture with a ratio of peat:vermiculite:perlite of 3:1:1. Regular irrigation and fertilization were conducted to maintain optimal growth conditions. Fertilization was performed with 5 grams per pot of NPK 15-15–15 compound fertilizer, applied once every 8 days after seedling emergence. Plants were grown in plastic pots with a diameter of 15 centimeters, filled with the sterilized potting mixture. The cultivation was carried out under controlled conditions: 28°C, a photoperiod of 16 hours of light/8 hours of darkness, and relative humidity of 50-60%. The substrate was sterilized prior to use to prevent microbial contamination.

Experimental treatments commenced at the three-leaf developmental stage, with triplicate biological replicates implemented throughout. Lepidopteran test species (*Spodoptera frugiperda, Ostrinia furnacalis, and Spodoptera exigua*) were obtained as laboratory-adapted colonies from the Engineering Technology Institute of Maize Breeding in Anhui Province. Larvae were maintained in climate-controlled chambers under standardized rearing conditions (25 ± 1°C, 60 ± 5% RH, 16h light cycle), fed artificial diet until reaching uniform 2nd-3rd instar developmental stages. Adult specimens received nutritional supplementation via 10% honey solution. All insect bioassays included triplicate biological replicates to ensure experimental robustness. The study employed a completely randomized design with three biological replicates per treatment. For lepidopteran infestation experiments, three-leaf stage maize plants were randomly assigned to four treatment groups: (1) control (no infestation), (2) *Spodoptera frugiperda* infestation, (3) *Ostrinia furnacalis* infestation, and (4) *Spodoptera exigua* infestation. Each treatment group contained 15 plants (3 plants per replicate). Infestation was performed using 2nd-instar larvae at a density of 5 larvae per plant.

### 2.2 Identification of the *ZmPAL*s pan gene family

The 26 maize pangenome datasets were retrieved from MaizeGDB following Hufford’s genomic framework (Hufford et al., 2021). The PAL-specific HMM profile (PF00320) was acquired from the InterPro database (Paysan-Lafosse et al., 2023). A dual-algorithm approach employing HMMER v3.3.2 and BLASTP identified candidate sequences containing PAL domains, with candidates selected using an E-value threshold <1e-10. Following sequence extraction and redundancy elimination, structural validation through SMART and functional annotation via UniProt confirmed definitive PAL family members across all pangenome assemblies (Bateman et al., 2021; Letunic et al., 2021) (Please refer to **Supplementary Table S1** for the URLs and access times of all the websites in Section 2).

### 2.3 Phylogenetic analysis of the *ZmPALs* pan gene family

Phylogenetic relationships among *ZmPAL*s proteins were reconstructed through comparative analysis with Arabidopsis and rice homologs. Reference PAL protein sequences from *Arabidopsis thaliana* and *Oryza sativa* were retrieved from NCBI and Phytozome13 databases (Pruitt et al., 2011; Goodstein et al., 2012). Multiple sequence alignment of maize, rice, and Arabidopsis PAL homologs was performed using the ClustalW algorithm, followed by phylogenetic tree construction via the neighbor-joining method in MEGA11 with 1000 bootstrap replicates. The resultant tree topology was visualized and annotated using Evolview to enhance interpretative resolution of evolutionary relationships (Subramanian et al., 2019).

### 2.4 Presence-absence variation in the *ZmPAL*s pan gene family

Presence-absence variation (PAV) profiling of *ZmPAL*s was conducted using genomic data from Hufford’s pangenome study. A custom script generated binary PAV matrices, which were subsequently processed for phylogenomic representations of 26 genotypes through the ggplot2 package in R. Presence/absence patterns across genotypes were visualized as heatmaps via the ComplexHeatmap R package, enabling systematic evaluation of PAL family genomic architecture heterogeneity.

### 2.5 Ka/Ks calculations for the *ZmPAL*s pan gene family

Coding sequences (CDS) and corresponding protein sequences of PAL family members were extracted from 26 maize genomes. Evolutionary selection pressures were quantified using KaKs_Calculator v2.0 to compute Ka/Ks ratios for each PAL homolog. A visualization pipeline integrating three R packages (ggridges, ggplot2, and pheatmap) generated comparative evolutionary profiles - ridgeline plots for full Ka/Ks distribution analysis and heatmaps specifically highlighting positive selection candidates (Ka/Ks>1). The evolutionary framework defined neutral mutations at Ka/Ks=1, positive selection at ratios >1, and purifying selection at ratios <1, enabling systematic detection of selection signatures across phylogenetic contexts (Li et al., 2023b).

### 2.6 Analysis of overlapping expression of structural variants of *ZmPAL*s

SV data and corresponding gene expression profiles across cultivars were retrieved from Hufford’s genomic resource, with B73 serving as the reference genome for structural variant calling. SV annotations were performed using ANNOVAR, followed by targeted extraction of PAL-associated structural variants. Correlation analyses between SV status and transcriptional activity of PAL members were conducted, with differentially expressed candidates visualized through histogram representations. Genome annotation files (GFF3 format) for all 26 maize lines were acquired from MaizeGDB to resolve structural configurations of significant PAL variants. Motif discovery in ZmPAL protein sequences was executed via the MEME Suite (v5.5.5) using default parameters except for specifying 10 motifs (Bailey et al., 2015). Final gene structure diagrams integrating motif patterns and genomic architectures were generated using TBtools, leveraging GFF annotations and motif prediction outputs for comparative visualization (Chen et al., 2023).

### 2.7 Analysis of cis-elements and structures of *ZmPAL*s from different maize

ZmPAL protein sequences from insect-resistant accession CML333 and reference genome B73 were submitted to the MEME Suite for comparative motif analysis, specifying 10 conserved motifs and generating WebLogo outputs to visualize sequence conservation patterns. Promoter regions (-2000 bp upstream) of ZmPALs were extracted from both genotypes for cis-element identification using PlantCARE database, prioritizing elements associated with stress responsiveness, developmental regulation, and hormonal signaling pathways (Rombauts et al., 1999).

### 2.8 Analyzing the *ZmPAL*s pan gene family using published RNA-Seq data

Publicly available RNA-seq datasets documenting maize responses to lepidopteran infestations (*Spodoptera frugiperda*: PRJNA675077, *Ostrinia furnacalis*: PRJNA772910, *Spodoptera exigua*: PRJNA625224) were retrieved from NCBI for expression profiling of *ZmPAL*s pan-genes (Tzin et al., 2017; Tang et al., 2021; Ye et al., 2022). Raw sequence quality was assessed using FastQC v0.12.1, followed by adapter removal and quality filtering through Trimmomatic v0.40. Processed reads were aligned to the B73_V5 reference genome using TBtools’ One-Step RNAseq plugin, implementing HISAT2 for alignment and StringTie for transcript quantification to generate FPKM expression matrices. Differential expression patterns were visualized as log_2_(RPKM+1)-transformed heatmaps using the ComplexHeatmap package (v2.6.2) in R v4.0.3, enabling cross-infestation comparative analysis of PAL transcriptional dynamics.

### 2.9 Expression properties of *ZmPAL*s genes under lepidopteran infestation

Lepidopteran infestation was performed by placing five 2nd-instar larvae (starved for 4 hr) on the abaxial surface of the third fully expanded leaf using a fine brush. Larvae were confined to individual plants using mesh bags (30×40 cm, 120-mesh) to prevent escape and ensure controlled feeding. Infestation duration was standardized to 24 hours for all experiments, after which larvae were removed and leaf damage was quantified. Leaf samples were collected from maize plants at 0h (uninfested control), 4h, 12h, and 24h post-infestation by lepidopteran larvae (*Spodoptera frugiperda, Ostrinia furnacalis, Spodoptera exigua*). Total RNA was isolated using the RNAprep Pure Plant Kit (TIANGEN, Beijing, China) following manufacturer protocols, with subsequent cDNA synthesis performed via HiscriptII Reverse Transcriptase (Vazyme, Nanjing, China). qRT-PCR primers targeting *ZmPAL*s were designed using Primer 6 software, with maize elongation factor 1-alpha (EF-1α) serving as the endogenous control (Ye et al., 2012) (**Supplementary Table S2**). Amplification reactions (20μL) containing 5μL cDNA template, 0.1μM primers, and SYBR Green Master Mix were conducted in triplicate on an ABI ViiA 7 system under standardized thermal cycling conditions: 95°C for 30 sec, 40 cycles of 95°C/10 sec and 60°C/30 sec, followed by melt curve analysis (95°C/15 sec, 60°C/60 sec, 95°C/15 sec). Melting curve profiles and cycle threshold (Ct) values were acquired using QuantStudio™ Software v1.6.1, with relative expression quantified through the 2^−ΔΔCt^ method (Livak and Schmittgen, 2001). All data were analyzed using SPSS 26.0 (IBM Corp.). One-way ANOVA followed by Tukey’s HSD test was employed for multiple comparisons between treatment groups. For temporal expression patterns, two-way ANOVA was used to examine the effects of time and treatment. Data are presented as mean ± standard error (SE) of three biological replicates, with statistical significance set at p < 0.05. Data visualization was performed using GraphPad Prism 8 software (Berkman et al., 2018). All qRT-PCR reactions were performed in triplicate for each sample.

## 3 Results

### 3.1 Identification and phylogenetic analysis of the *ZmPAL*s pan-genome

**Figure 1:**
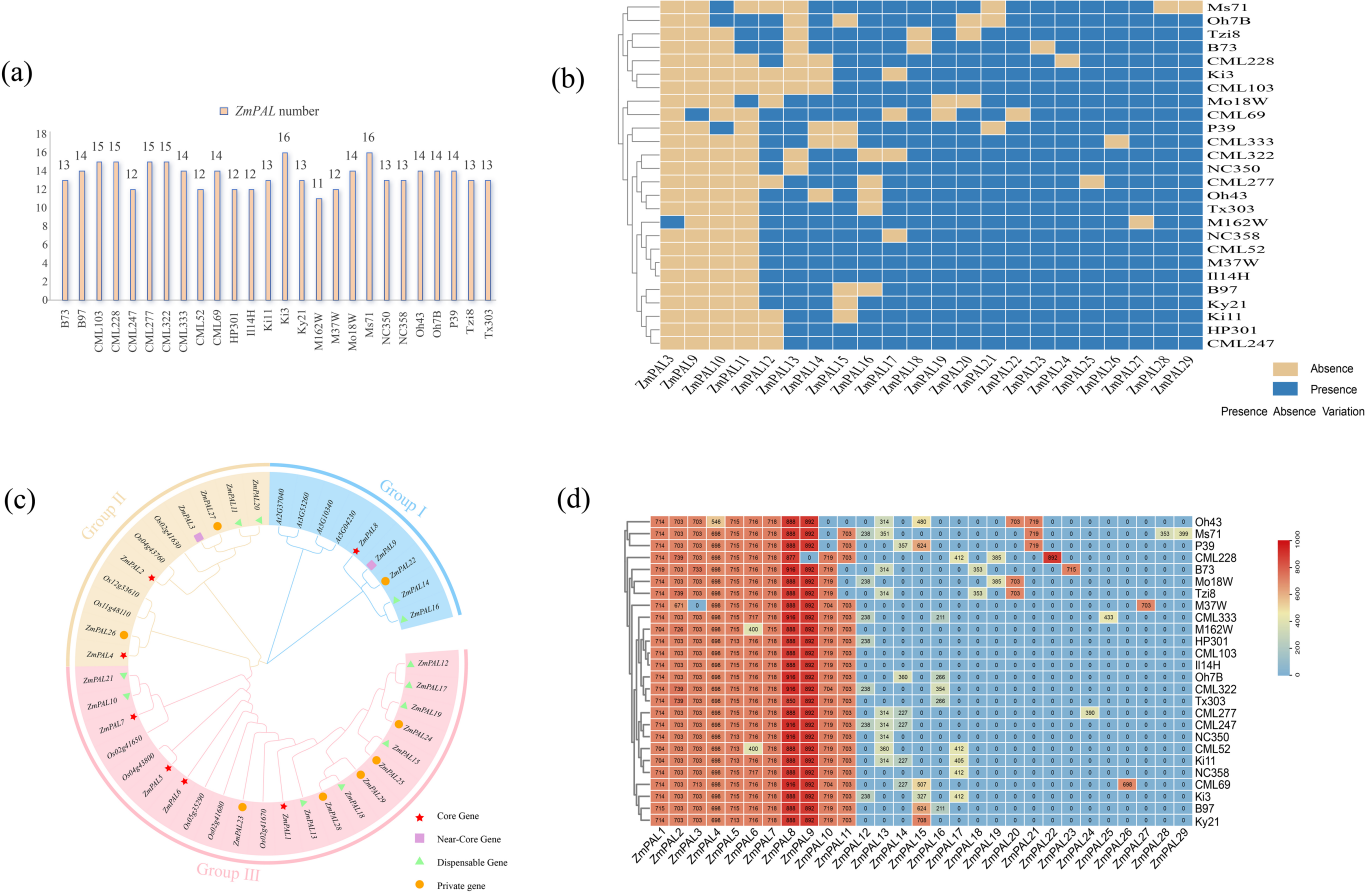
Identification and phylogenetic analysis of *ZmPALs* in pan-genome. **(a)** Number of *ZmPALs*. **(b)** Heatmap of the presence and absence of 21 *ZmPALs* in 26 maize varieties except for the core genes. **(c)** Phylogenetic tree of PALs from Arabidopsis and Maize. **(d)** Heatmap of *ZmPAL*s protein length.

A total of 29 *ZmPAL*s family members were identified across 26 maize pan-genome assemblies. Comparative analysis using B73 as the reference genome revealed marked disparities in both gene count and protein sequence length among different maize lines (**Figure 1a**). The Ki3 and MS71 lines exhibited the highest PALs content with 16 members each, while M162W showed the minimal count of 11 members. Structural classification of *ZmPAL*s demonstrated a composition of 7 core genes, 2 near-core genes, 12 non-core genes, and 8 line-specific private genes (**Figure 1b**). The identification of 29 *ZmPAL*s across 26 maize genomes revealed four distinct categories: (1) 7 core genes present in all accessions, likely maintaining essential functions in phenylpropanoid metabolism; (2) 2 near-core genes absent in only 1–2 lines, suggesting conserved but potentially specialized roles; (3) 12 non-core genes showing intermediate frequencies (present in 3–24 genomes), which may contribute to lineage-specific adaptations; and (4) 8 private genes unique to single accessions, representing recent evolutionary innovations or deletions in other lines. This distribution pattern reflects the dynamic evolutionary history of the *ZmPAL* family, where core genes preserve fundamental functions while non-core and private genes may enable metabolic diversification and environmental adaptation. Particularly, the substantial proportion of non-core and private genes (20/29, ~69%) highlights the remarkable genetic plasticity of phenylpropanoid pathways in maize, potentially facilitating rapid responses to biotic stresses like lepidopteran herbivory across different environments.

Protein length comparisons revealed that *ZmPAL8* (core) displayed the longest sequence, followed by *ZmPAL7*. The near-core *ZmPAL9* maintained consistent protein length in 25 lines despite its absence in CML228. Among non-core genes, *ZmPAL21* exhibited the longest average protein length compared to the shortest *ZmPAL12*. Substantial variation in protein lengths was observed across other family members (**Figure 1d**). Comprehensive data for all PAL family members are provided in **Supplementary Table S3**.

Phylogenetic analysis utilizing protein sequences from maize, rice, and Arabidopsis established three distinct subfamilies following the classification by Wu et al. (**Figure 1c**). Subfamily III contained the highest number of *ZmPAL*s (17 members), followed by Subfamily II with 7 members, while Subfamily I exhibited the smallest contingent (5 members). Core, non-core, and private genes predominantly accumulated in Subfamily III, though near-core genes were notably absent from this subfamily. Arabidopsis PALs were exclusively clustered in Subfamily I, whereas rice PALs distributed across both Subfamilies II and III. This evolutionary pattern demonstrates closer phylogenetic relationship between maize and rice compared to their divergence from Arabidopsis.

### 3.2 *ZmPAL*s are subject to different selection pressures among maize varieties

**Figure 2:**
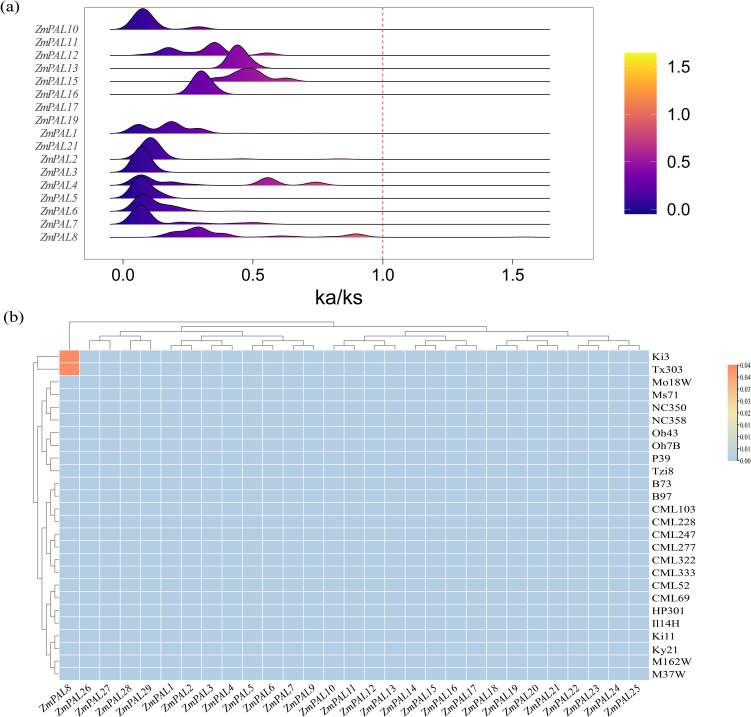
Ka/Ks values of ZmPALs. **(a)** Distribution of Ka/Ks values of *ZmPAL* for 26 maize varieties; **(b)** heat map of the frequency of occurrence of different maize varieties with Ka/Ks ratios > 1 in each PAL.

The Ka/Ks ratio served as a critical evolutionary indicator to investigate selection pressures on PAL family members across 26 maize genomes. Comprehensive calculation of Ka/Ks values revealed that except for private genes (uncalculable ratios), only *ZmPAL8* exhibited a ratio exceeding 1, while most members maintained ratios below this threshold (**Figure 2a**). Distinct positive selection signals were detected for *ZmPAL8* in Ki3 and Tx303 lines, contrasting with purifying selection patterns in other genes. Heatmap analysis of ratios >1 confirmed *ZmPAL8* as the sole gene demonstrating elevated Ka/Ks values, indicative of sustained selective pressure during maize evolution (**Figure 2b**).

### 3.3 Effect of SV on gene expression, structure and motifs of *ZmPAL*s

**Figure 3:**
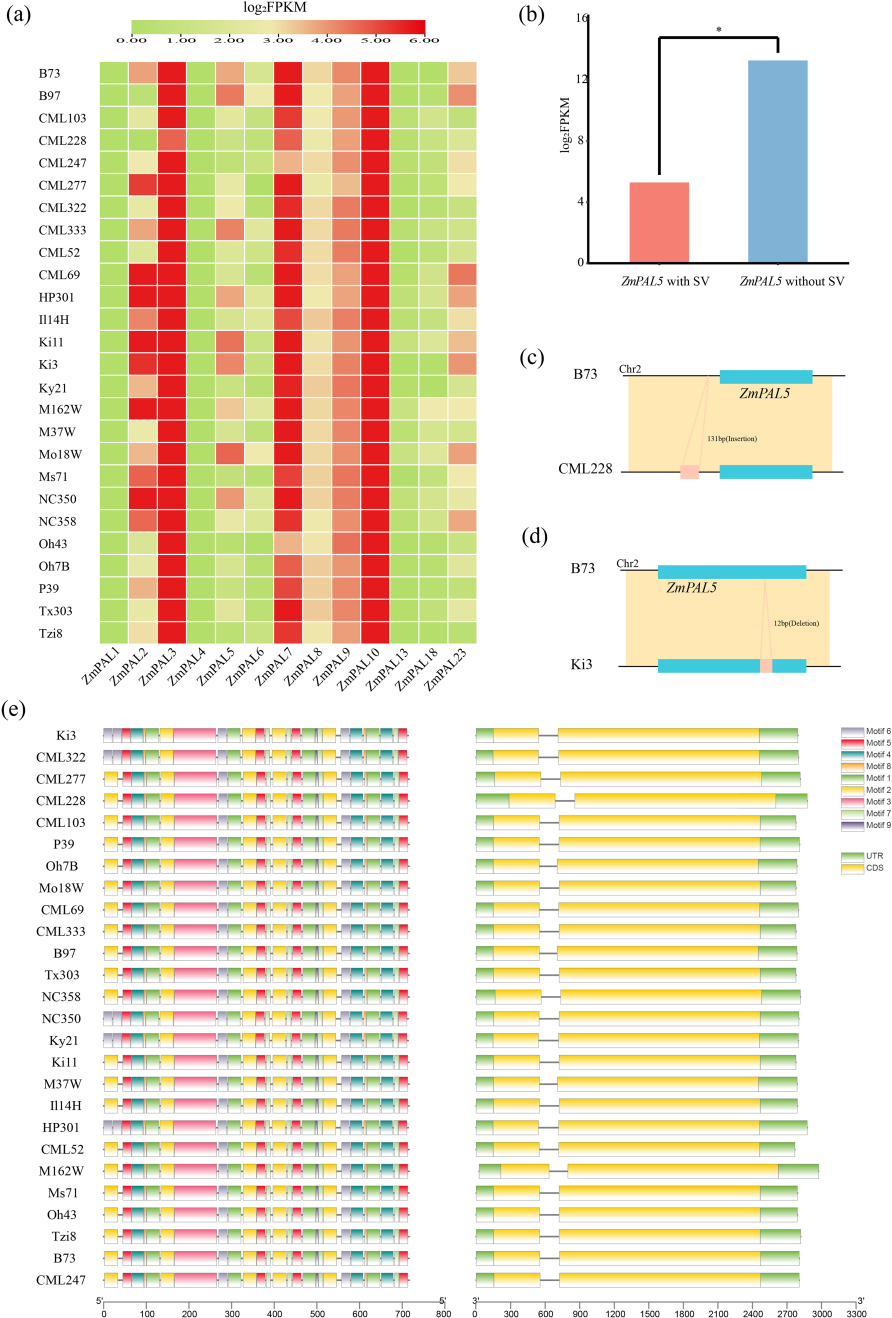
SV affects the expression, structure and motifs of 26 maize genomes. **(a)** SV on the expression of 26 maize pan-genomes; **(b)** SV significantly affects the expression of *ZmPAL5* (*: P<0.05); **(c)** effect of SV insertion on *ZmPAL5*; **(d)** effect of SV deletion on *ZmPAL5*; **(e)** structure and motifs of *ZmPAL5* in the maize pan-genome.

Structural variation (SV) analysis of PAL family members revealed distinct expression patterns through Pearson correlation coefficients comparing SV-overlapping and non-overlapping genes. Expression profiling demonstrated elevated transcript levels for *ZmPAL3, ZmPAL7*, and *ZmPAL10*, while *ZmPAL1* and *ZmPAL13* showed complete transcriptional silence (**Figure 3a**). Notably, *ZmPAL5* exhibited significant differential expression between SV-present and SV-absent conditions (p<0.05), demonstrating substantial SV-mediated regulation of its transcriptional activity (**Figure 3b**).

Genomic analysis identified 196 structural variations (SVs) overlapping with 29 *ZmPAL*s loci and their flanking 2-kb regulatory regions. Comparative profiling against the B73 reference genome revealed SVs predominantly manifested as insertions (**Figure 3c**) and deletions (**Figure 3d**). Notably, line CML228 exhibited a 131-bp upstream insertion, while Ki3 displayed a 12-bp intragenic deletion. Comparative structural mapping of *ZmPAL5* across 26 genomes demonstrated conserved gene architecture and conserved domains identical to the B73 reference, with no substantial structural divergences observed (**Figure 3e**).

### 3.4 Cis-elements and structural analysis of *ZmPAL*s

**Figure 4:**
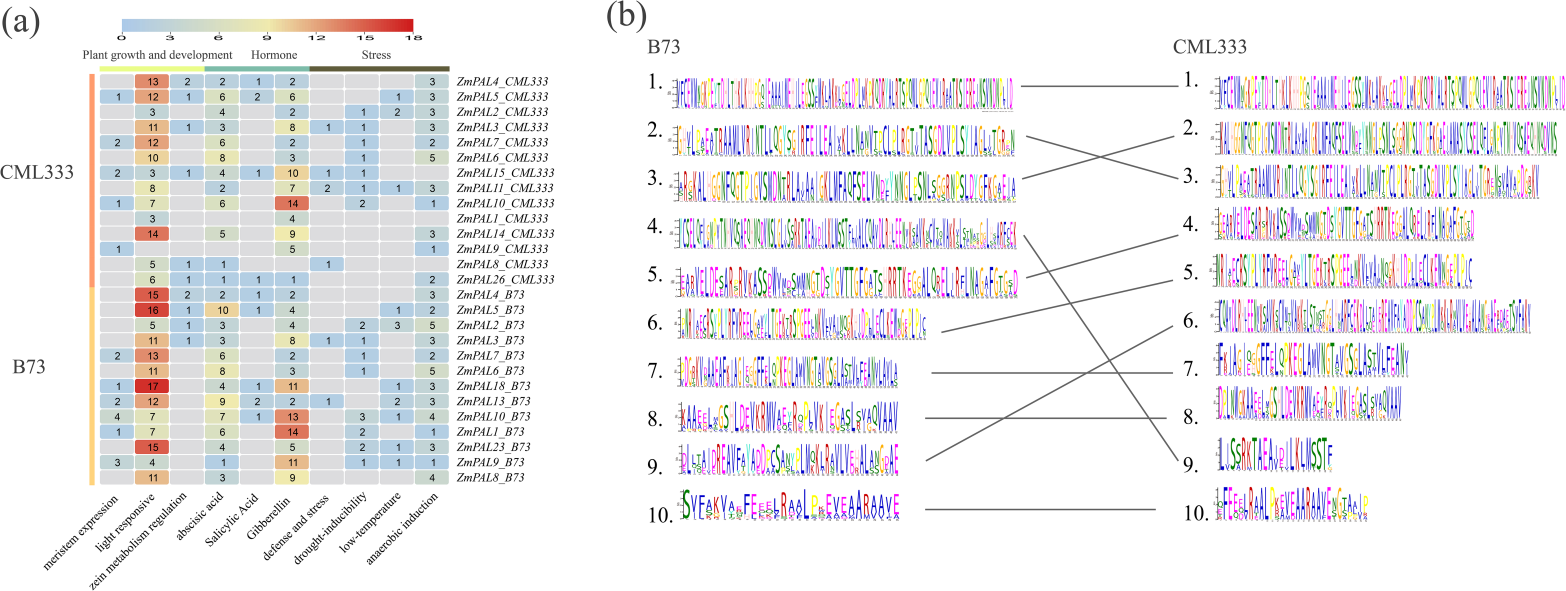
Cis-elements and structural analysis of *ZmPALs*. **(a)** Number and functional classification of cis-acting elements in the B73 and CML333 genomes. **(b)** Web logos of *ZmPALs* in CML333 and B73 are shown on the left and right, respectively. Web logos connected by lines indicate that they correspond. webLogos are arranged in the order of E-values.

Comparative analysis of cis-acting elements in *ZmPAL*s promoters was conducted between CML333 and B73, focusing on hormone responsiveness, plant growth/development, and stress-related regulatory motifs (**Figure 4a**). The B73 genome exhibited greater cis-element diversity compared to CML333. Hormonal response elements for abscisic acid, gibberellin, and salicylic acid were prevalent across most ZmPAL promoters. Light-responsive elements were ubiquitously present except in *ZmPAL9*_CML333. Stress-inducible motifs including low-temperature responsiveness, anaerobic induction, and drought-inducibility were systematically identified. These findings suggest that *ZmPAL*s may orchestrate maize growth, development, and stress adaptation through combinatorial regulation by diverse promoter cis-elements.

Based on the conserved motifs of ZmPALs in the reference genomes B73 and CML333, a conserved domain alignment diagram was generated (**Figure 4b**). The results indicated that all ten conserved domains identified in the CML333 genome corresponded to those in the B73 reference genome. However, the amino acids within the corresponding conserved domains of each group did not exhibit complete alignment, a phenomenon potentially attributable to selective pressures affecting the conserved domains of ZmPALs.

### 3.5 Transcriptome analysis of the *ZmPAL*s gene family under stress in different lepidopteran insects

**Figure 5:**
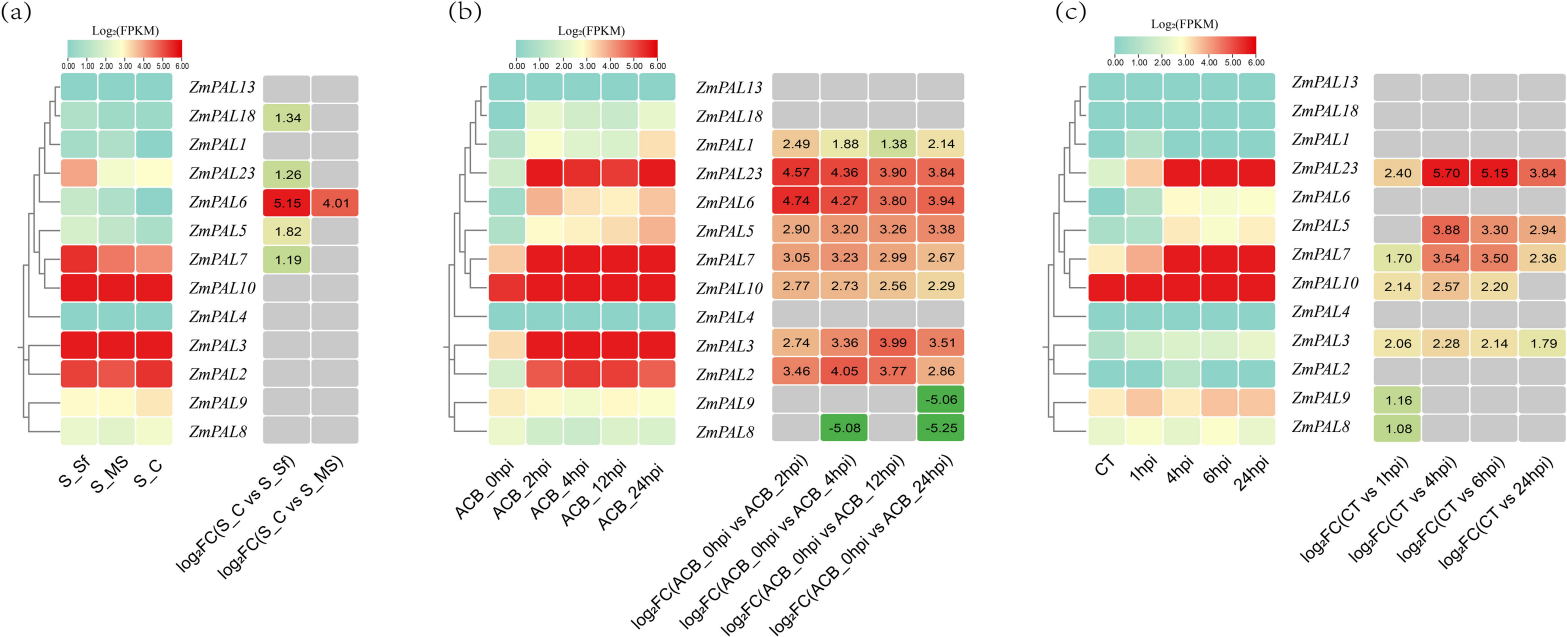
Expression profiles of *ZmPALs* under Lepidopteran herbivory. **(a)** *Spodoptera frugiperda* challenge: S_C (Control), S_Sf (Third-day larval feeding), S_MS (Mechanical wounding control). **(b)** Dynamic response to *Ostrinia furnacalis* infestation: Temporal expression patterns at 0, 4, 12, and 24 hours post-infestation (hpi). **(c)** *Spodoptera exigua* exposure: CT (Untreated control) and stress responses at 1, 4, 6, 24 hpi. Note: left matrix shows normalized FPKM values, while the right matrix illustrates differential expression through log2(fold change) values (|FC|≥1 threshold), with red and green color gradients denoting upregulation and downregulation respectively.

Transcriptomic re-analysis of published datasets (PRJNA675077: *Spodoptera frugiperda*; PRJNA772910: *Ostrinia furnacalis*; PRJNA625224: *Spodoptera exigua*) using the B73_V5 reference genome revealed conserved expression patterns among *ZmPAL*s pan-family members under Lepidopteran herbivory (**Figure 5**). Two members (*ZmPAL4* and *ZmPAL13*) exhibited transcriptional silence across all stress conditions, while five genes (*ZmPAL2, ZmPAL3, ZmPAL7, ZmPAL10* and *ZmPAL23*) demonstrated elevated expression profiles. The pan-family displayed coordinated transcriptional responses to these biotic stressors, suggesting functional conservation in insect defense mechanisms.

Lepidopteran-specific induction patterns revealed distinct regulatory responses among *ZmPAL*s members. *Spodoptera frugiperda* infestation (**Figure 5a**) triggered significant upregulation of five genes (*ZmPAL5, ZmPAL6, ZmPAL7, ZmPAL18* and *ZmPAL23*), with *ZmPAL6* showing the most pronounced induction. *Ostrinia furnacalis* challenge (**Figure 5b**) upregulated eight *ZmPAL*s (*ZmPAL1, ZmPAL2, ZmPAL3, ZmPAL5, ZmPAL6, ZmPAL7, ZmPAL10* and *ZmPAL23*), particularly *ZmPAL6* and *ZmPAL23*, while downregulating *ZmPAL8* and *ZmPAL9*. *Spodoptera exigua* exposure (**Figure 5c**) elevated seven transcripts (*ZmPAL3*, *ZmPAL5*, *ZmPAL7*, *ZmPAL8*, *ZmPAL9*, *ZmPAL10* and *ZmPAL23*), with *ZmPAL23* demonstrating the highest induction magnitude.

### 3.6 Analysis of the expression pattern of the *ZmPAL*s gene family after FAW infestation

**Figure 6:**
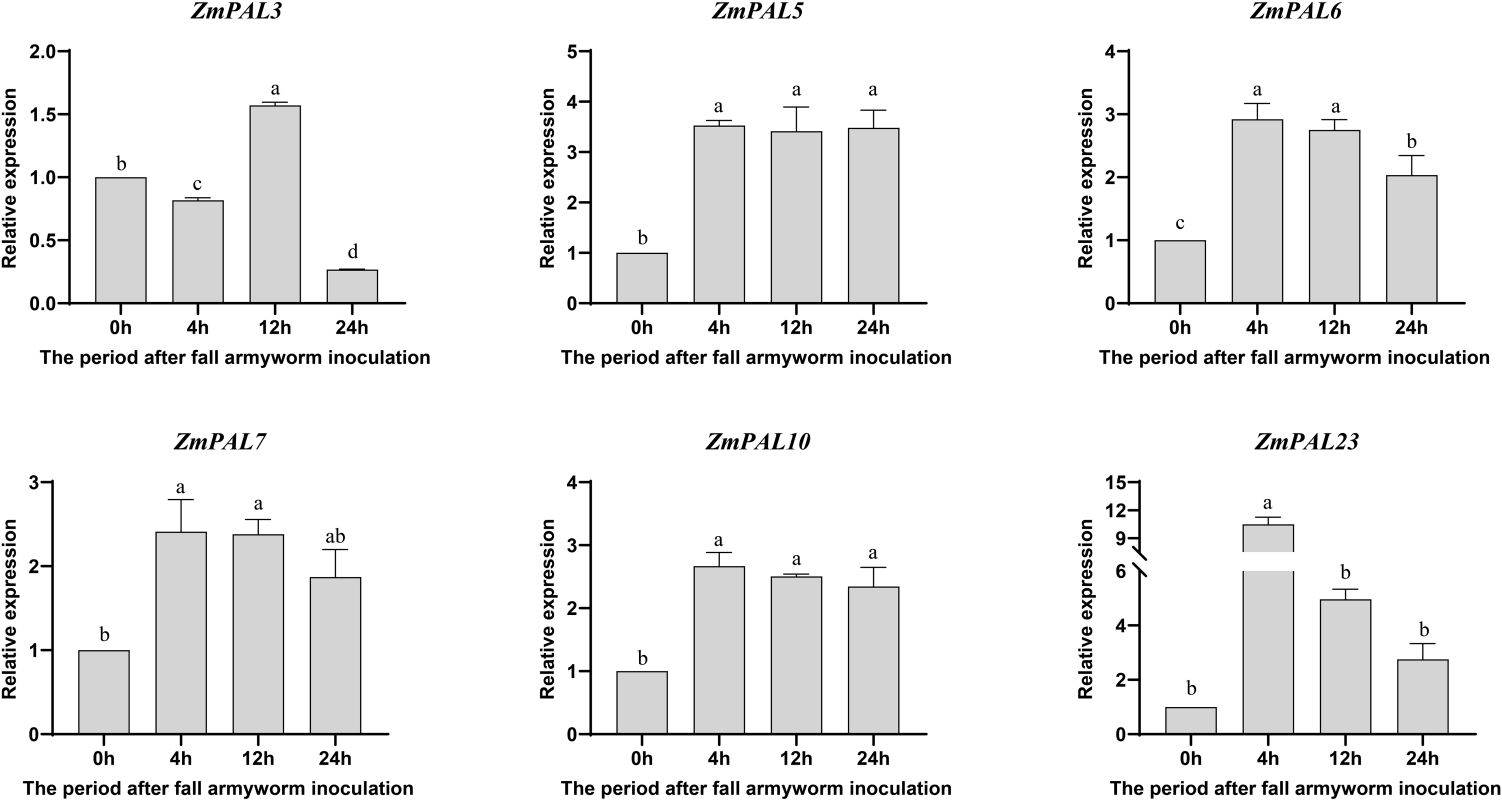
Expression pattern of *ZmPALs* pan gene family after FWA infestation. The time of infestation was set to four time points (0, 4, 12 and 24 hpi after infestation), with 0h as a control. Significant differences are indicated by lower case letters (*p* < 0.05).

To elucidate *ZmPAL*s pan-family functions in maize defense against fall armyworm (FAW), a time-course induction experiment was conducted in B73 plants. Quantitative PCR analysis revealed dynamic temporal expression patterns of six *ZmPAL*s (*ZmPAL3*, *ZmPAL5*, *ZmPAL6*, *ZmPAL7*, *ZmPAL10* and *ZmPAL23*) at 0, 4, 12, and 24 hours post-infection (hpi) (**Figure 6**). All examined genes exhibited FAW-responsive regulation, with significant upregulation peaking during early to mid-stages (4–12 hpi). Transcript levels subsequently declined to basal levels by 24 hpi, indicating transient induction kinetics characteristic of biotic stress responses.

Five *ZmPAL*s members (excluding *ZmPAL3*) exhibited significant induction across FAW infestation stages. *ZmPAL5* and *ZmPAL6* peaked at 12 hpi, whereas *ZmPAL23* showed an early expression surge at 4 hpi (**Figure 6**). The transient *ZmPAL3* upregulation at 12 hpi coincided with defense signaling activation, while its downregulation at 4 hpi and 24 hpi suggests dynamic transcriptional reprogramming coordinating anti-herbivore responses. These differential patterns implicate *ZmPAL*s in FAW defense through temporal-specific regulation, potentially mediated by phased induction of defense-related signaling cascades. The observed temporal divergence in gene activation (early vs mid-phase peaks) reflects functional specialization within the PAL regulatory network during biotic stress adaptation.

### 3.7 Expression pattern analysis of *ZmPAL*s gene family after ACB infestation

**Figure 7:**
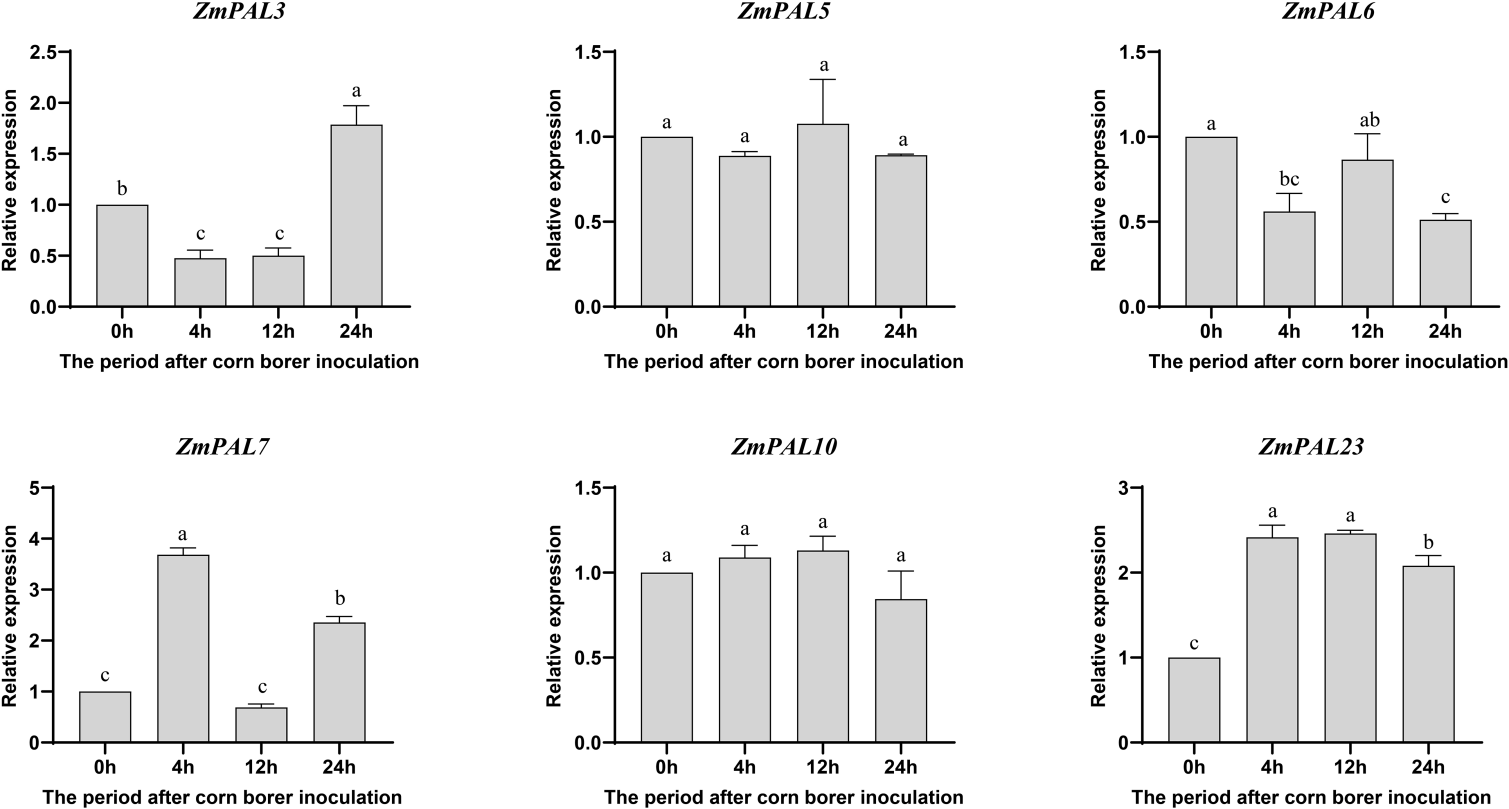
Expression pattern of *ZmPALs* pan gene family after ACB infestation. The time of infestation was set to four time points (0, 4, 12 and 24 hpi after infestation), with 0h as a control. Significant differences are indicated by lower case letters (*p* < 0.05).

Functional investigation of *ZmPAL*s pan-family members under Asian corn borer (ACB) infestation revealed temporally differentiated expression dynamics in B73 (**Figure 7**). *ZmPAL5* and *ZmPAL10* showed no significant expression alterations, suggesting limited involvement in ACB response. Notably, *ZmPAL7* exhibited significant upregulation at 4 hpi, indicating potential early defense activation, while *ZmPAL3* demonstrated delayed induction peaking at 24 hpi, possibly mediating late-phase resistance mechanisms. *ZmPAL6* displayed consistent downregulation across all timepoints, suggesting suppression during ACB challenge. In contrast, *ZmPAL23* demonstrated sustained induction throughout infestation stages, highlighting its central regulatory role. These divergent expression profiles reflect functional specialization within the *ZmPAL*s network during ACB defense, with specific members orchestrating phase-specific resistance strategies. The identification of temporally regulated PAL isoforms provides both mechanistic insights into maize-insect interactions and potential genetic targets for breeding ACB-resistant varieties.

### 3.8 Expression pattern analysis of the *ZmPAL*s gene family after BAW infestation

**Figure 8:**
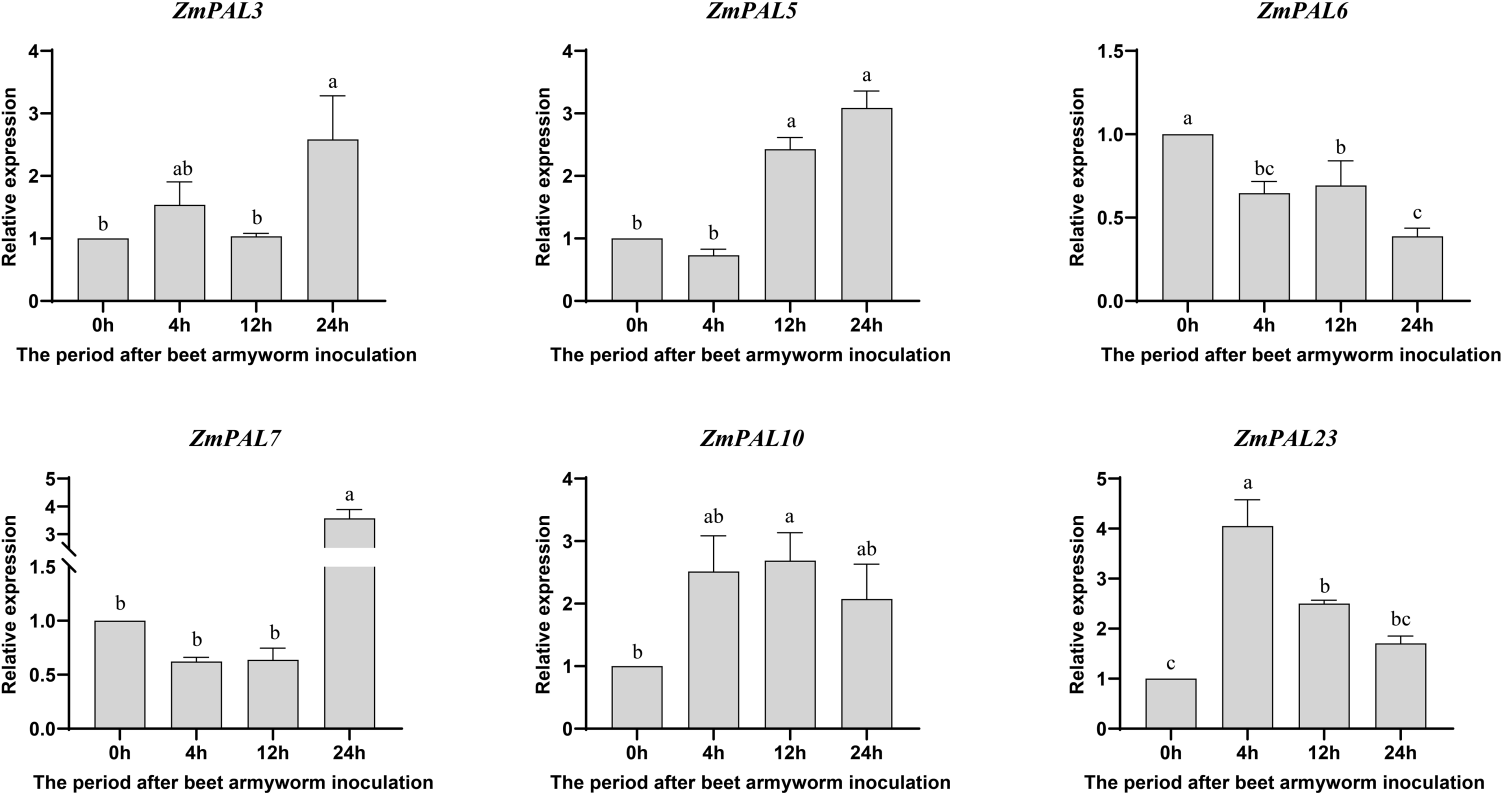
Expression pattern of *ZmPALs* pan gene family after BAW infestation. The time of infestation was set to four time points (0, 4, 12 and 24 hpi after infestation), with 0h as a control. Significant differences are indicated by lower case letters (*p* < 0.05).

qRT-PCR analysis of *ZmPAL*s expression in B73 following beet armyworm (BAW) infestation revealed temporally stratified transcriptional dynamics across four timepoints (0, 2, 12, 24 hpi) (**Figure 8**). All responsive *ZmPAL*s exhibited phased induction patterns: ZmPAL23 showed marked upregulation at 4 hpi, while *ZmPAL7* peaked at 24 hpi. Early response genes (*ZmPAL10* and *ZmPAL23*) demonstrated significant induction by 4 hpi, suggesting initiation of primary defense mechanisms. Mid-phase induction (12 hpi) involved *ZmPAL5*, *ZmPAL10*, and *ZmPAL23*, potentially coordinating intermediate defense signaling. Late-stage upregulation (24 hpi) characterized *ZmPAL3* and *ZmPAL7*, with *ZmPAL10* maintaining sustained 2–3 fold induction post-4 hpi. Contrastingly, *ZmPAL6* displayed consistent downregulation, indicating negative regulatory functions. The observed temporal divergence in PAL activation suggests stage-specific defensive roles, particularly mid-phase induction potentially mediating defense pathway modulation. This dynamic transcriptional reprogramming reflects ongoing plant-insect interplay through successive defense phases.

## 4 Discussion

The PAL gene family in maize was first identified by the B73 reference genome (B73 RefGen_v3) (Lv et al., 2016). Traditional gene family analyses are usually based on a single reference genome, and given that a single reference genome is insufficient to capture the full extent of genetic diversity within a species, determining the presence or absence of genes in multiple reference genomes becomes challenging (Golicz et al., 2016; Silva-Arias et al., 2024). Recently, a pan-genome of 26 high-quality maize genomes containing genes missing from the reference genome has been published, which provides a more precise assembly and annotation than the B73 reference genome when compared to reference genome-based gene family analyses (Schnable et al., 2009; Hirsch et al., 2014). In this study, 29 *ZmPAL*s were identified using the maize pan-genome, including 7 core genes, 2 near-core genes, 12 non-core genes and 8 private genes. This is an increase of 16 genes compared to the 13 genes identified in the reference genome (Wu et al., 2020). PAV analysis showed that only 7 of the 29 gene family members were consistently present in all maize varieties and the core genes were distributed in all three subgroups, and these core genes may play important regulatory roles in maize growth and development. In addition, the remaining genes are not universally absent in all varieties, thus ensuring genomic complementarity between varieties (Lin et al., 2024). *ZmPAL28* and *ZmPAL29* are present only in Ms71 and regulate traits that are unique to this line. Despite the variation in the number of *ZmPAL*s, total gene expression showed no correlation. This may be due to the fact that some members of the gene family have similar functions and can compensate each other to maintain normal physiological processes (Iohannes and Jackson, 2023).

Structural variations (SVs), encompassing deletions, insertions, inversions, and translocations, represent critical genetic modifications that can alter gene architecture, conserved domains, and transcriptional regulation (Tang, 2020; Kalakoti et al., 2025). SVs substantially influence crop resilience to biotic and abiotic stressors, as exemplified by the SNP-465 variant in *ZmICE1*’s promoter region modulating cold tolerance through transcriptional reprogramming (Jiang et al., 2022), and *ZmBGLU17*’s promoter SV enhancing resistance against Phytophthora pathogens and Asian corn borer (Liu et al., 2023a). This study identified SV-mediated regulation of *ZmPAL5* expression warranting mechanistic investigation. Comparative analysis revealed a 131-bp insertion in CML333’s upstream region disrupted coding sequence alignment, resulting in complete *ZmPAL5* transcriptional silencing. Conversely, the 12-bp intragenic deletion in Ki3’s non-coding region exhibited minimal impact on *ZmPAL5* functionality, highlighting the positional sensitivity of structural variations in gene regulation. The Ka/Ks analysis in this study revealed that *ZmPAL8* underwent positive selection in both Ki3 and Tx303, whereas the other *ZmPAL*s experienced purifying selection (**Figure 2**). This suggests that *ZmPAL8* may contribute to local adaptation by enhancing phenylpropanoid biosynthesis related to defense mechanisms. In contrast, *ZmPAL7* and *ZmPAL10* exhibited conservative Ka/Ks ratios across different varieties, reflecting their non-redundant roles in fundamental metabolism. This differential selective pressure is consistent with the observed PAVs and SVs, highlighting how genetic diversity shapes the functional specialization of the *ZmPAL* family.

The enhanced insect resistance in CML333 correlates with its specialized chemical defense system, particularly benzoxazinoid (Bx) biosynthesis known for insect deterrence and toxicity (Kumar, 2002; Ni et al., 2008b). Comparative cis-regulatory element analysis of *ZmPAL*s promoters revealed greater element diversity in B73 versus CML333. However, evolutionary optimization in CML333 under prolonged natural and artificial selection pressures appears to have enhanced cis-element functional efficiency, potentially enabling rapid anti-herbivore gene activation. This regulatory refinement complements CML333’s predominant Bx-mediated defense strategy, contrasting with B73’s reliance on Bt protein expression and volatile organic compound release (Ni et al., 2008a; Matova et al., 2020). The observed inverse relationship between cis-element quantity and functional efficacy suggests evolutionary trajectory differences in pest resistance mechanisms between these genotypes. Such specialization aligns with ecological adaptation theory, where sustained pest pressure drives optimization of specific defense pathways rather than generalized regulatory complexity.

Plant insect resistance represents a pivotal adaptive trait in maize defense against Lepidopteran pests, mediated through intricate metabolic network regulation (Peterson et al., 2016; War et al., 2018; Prasanna et al., 2022). Studies demonstrate herbivory-induced activation of secondary metabolic pathways, with the phenylpropanoid pathway serving as a central hub for biosynthesis of lignin, phenolic compounds, and flavonoids (Singer et al., 2003; Singh et al., 2021; Dong et al., 2024). These metabolites function synergistically through physical barrier formation and phytochemical accumulation to disrupt insect feeding and development (Koul, 2008; Gupta and Roy, 2021). In *Spodoptera frugiperda*-infested maize, the *gl8* cuticular wax-deficient mutant exemplifies metabolic compensation mechanisms, exhibiting significant upregulation of phenylpropanoid-related genes concurrent with enhanced jasmonic acid (JA) signaling and benzoxazinoid accumulation (Liu et al., 2022). This metabolic reprogramming reveals plants’ adaptive strategies in balancing physical defenses (cuticular layers) with chemical defenses (specialized metabolites) (Ahmad et al., 2024). The preferential activation of phenylpropanoid metabolism under compromised physical barriers suggests its critical role as a compensatory defense mechanism against herbivory pressure, potentially through toxin-mediated larval growth inhibition.

Phenylalanine ammonia-lyase the rate-limiting enzyme in phenylpropanoid metabolism (Kong, 2015), serves as a central regulator in plant anti-herbivore responses (Li et al., 2023a; Zhu et al., 2024). Studies demonstrate that maize *ZmPAL*s members (e.g., *ZmPAL5*) mediate drought-induced resistance through catalytic production of trans-cinnamic acid from phenylalanine (Amorim-Silva and Botella, 2025), stimulating lignin biosynthesis to fortify cell wall rigidity against insect mandible penetration (Al-Khayri et al., 2023). This mechanism aligns with findings by Rojanaridpiched et al. linking lignification and silicon deposition to reduced leaf herbivory rates (Rojanaridpiched et al., 1984; Jiménez-Galindo et al., 2019), while Williams’ research implicates hemicellulose content in *Spodoptera frugiperda* resistance (Williams et al., 1998). PAL activity positively correlates with flavonoid phytoalexin accumulation, exemplified by PAL-mediated flavonoid biosynthesis disrupting lepidopteran gut microbiota homeostasis, thereby inducing larval developmental arrest and mortality (Sun et al., 2022). Notably, PAL functionality integrates with defense signaling networks through cross-talk with ethylene and jasmonic acid (JA) pathways (Yang et al., 2015; Li et al., 2019). For instance, the maize expansin *EXP-A20* enhances PAL-dependent lignification via ethylene biosynthesis gene *ACO31* activation, establishing multi-layered defense architecture against herbivores (Batool et al., 2024).

Reprocessed transcriptomic datasets from maize under Lepidopteran herbivory (*Spodoptera frugiperda*, *Ostrinia furnacalis*, *Spodoptera exigua*) using the B73_V5 reference genome revealed conserved transcriptional responses across ZmPALs family members. Two genes (*ZmPAL4* and *ZmPAL13*) exhibited complete transcriptional silence, while five members (*ZmPAL2*, *ZmPAL3*, *ZmPAL7*, *ZmPAL10* and *ZmPAL23*) demonstrated constitutively elevated expression profiles. Comparative analysis identified six candidate genes (*ZmPAL3*, *ZmPAL5*, *ZmPAL6*, *ZmPAL7*, *ZmPAL10* and *ZmPAL23*) with significant upregulation patterns relative to control conditions, suggesting their prioritized roles in insect defense signaling cascades. The conserved stress-responsive expression architecture across phylogenetically distinct Lepidopteran species implies evolutionary conservation of PAL-mediated defense mechanisms in maize.

qRT-PCR validation in B73 under Lepidopteran herbivory (*Spodoptera frugiperda, Ostrinia furnacalis, Spodoptera exigua*) revealed temporally stratified expression dynamics of *ZmPAL*s members. *ZmPAL7*, *ZmPAL10*, and *ZmPAL23* demonstrated marked induction during initial defense phases, while *ZmPAL10* and *ZmPAL23* maintained elevated expression through mid-late infestation stages. Intriguingly, *ZmPAL6* exhibited contrasting regulation - significant upregulation during FAW challenge versus downregulation under BAW stress, indicating insect-specific transcriptional reprogramming. *ZmPAL23* emerged as a central regulator, showing sustained upregulation across all Lepidopteran stressors and timepoints. These findings provide novel insights into PAL-mediated defense mechanisms against herbivores, revealing both conserved and species-specific regulatory strategies. The temporally coordinated activation patterns suggest hierarchical gene network functionality, where early-responsive genes initiate defense signaling while persistent effectors maintain resistance throughout infestation phases. This temporal specialization in PAL isoform activation advances understanding of maize’s molecular adaptation to Lepidopteran pressures.

While this study provides valuable insights into expression patterns and potential functions of *ZmPAL*s pan-family members in maize, several constraints warrant consideration. The analysis primarily relies on transcriptomic data, necessitating integrated multi-omics analyses encompassing proteomic and metabolomic dimensions to fully characterize PAL-mediated defense mechanisms. Although qRT-PCR validated expression profiles of selected genes, empirical validation through transgenic approaches or CRISPR-based functional genomics remains essential to establish causal relationships. These findings establish a foundational framework for understanding PAL-mediated Lepidopteran resistance while highlighting key knowledge gaps in isoform-specific regulatory networks. Subsequent investigations should prioritize mechanistic studies elucidating temporal coordination between PAL isoforms and downstream defense pathways. Such efforts will advance molecular breeding strategies aimed at enhancing maize resilience through optimized PAL-mediated defense architectures.

## 5 Conclusions

Pan-genomic comparative analysis identified 29 *ZmPAL*s family members across 26 maize genomes, comprising 7 core, 2 near-core, 12 non-core, and 8 private genes. Phylogenetic classification revealed three evolutionary subgroups, with Group III containing the largest membership (17 genes). Evolutionary pressure analysis demonstrated positive selection acting on *ZmPAL8* in specific accessions, while purifying selection dominated other members. SV analysis revealed SV-mediated regulation of *ZmPAL5* expression, where a 131-bp upstream insertion in CML333 resulted in transcriptional silencing.

Transcriptomic profiling under Lepidopteran herbivory (*Spodoptera frugiperda, Ostrinia furnacalis, Spodoptera exigua*) revealed temporally dynamic expression patterns. qRT-PCR validation confirmed stage-specific regulation: *ZmPAL7*, *ZmPAL10*, and *ZmPAL23* showed early-phase induction, while *ZmPAL10* and *ZmPAL23* maintained elevated expression through mid-late infestation stages. Notably, *ZmPAL6* exhibited contrasting regulation - upregulation during *Spodoptera frugiperda* challenge versus downregulation under *Spodoptera exigua* stress, indicating insect-specific transcriptional responses. *ZmPAL23* demonstrated sustained upregulation across all temporal phases, suggesting its central regulatory role in coordinated defense mechanisms. These findings delineate temporal specialization and functional divergence within *ZmPAL*s, providing mechanistic insights into phenylpropanoid pathway regulation during insect resistance.

## Data Availability

The original contributions presented in the study are included in the article/**Supplementary Material**. Further inquiries can be directed to the corresponding author/s.
